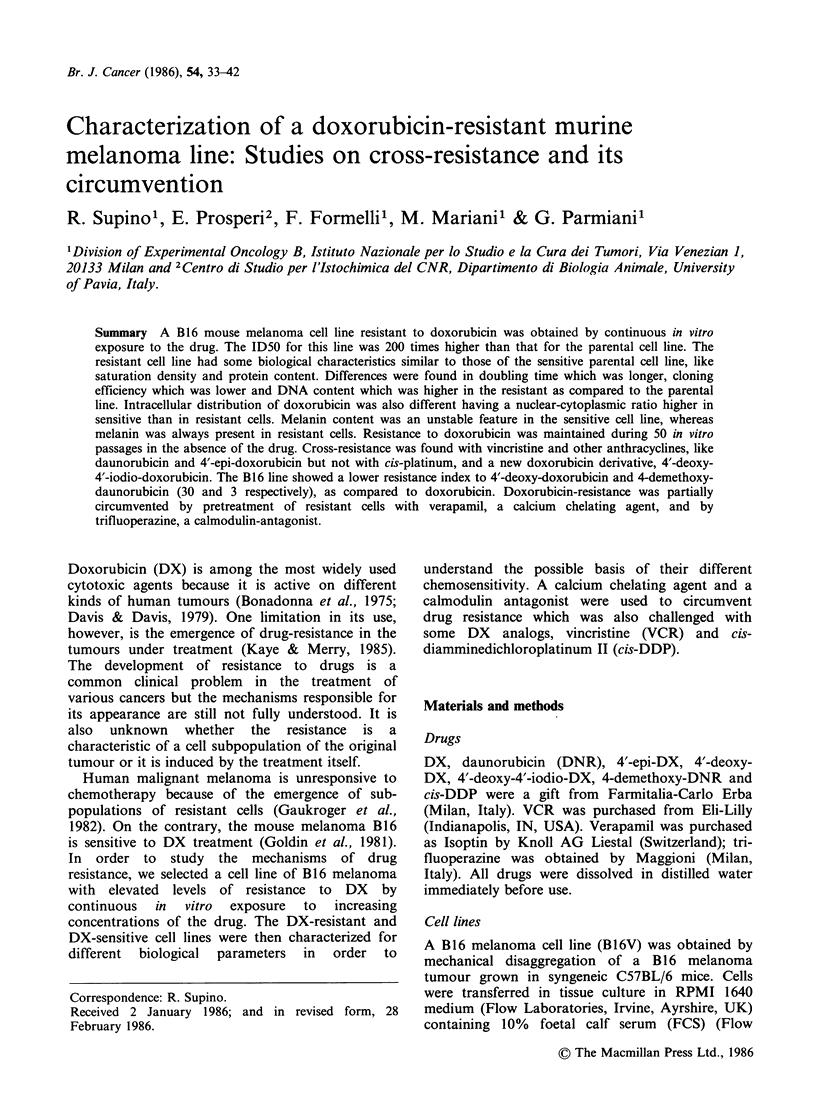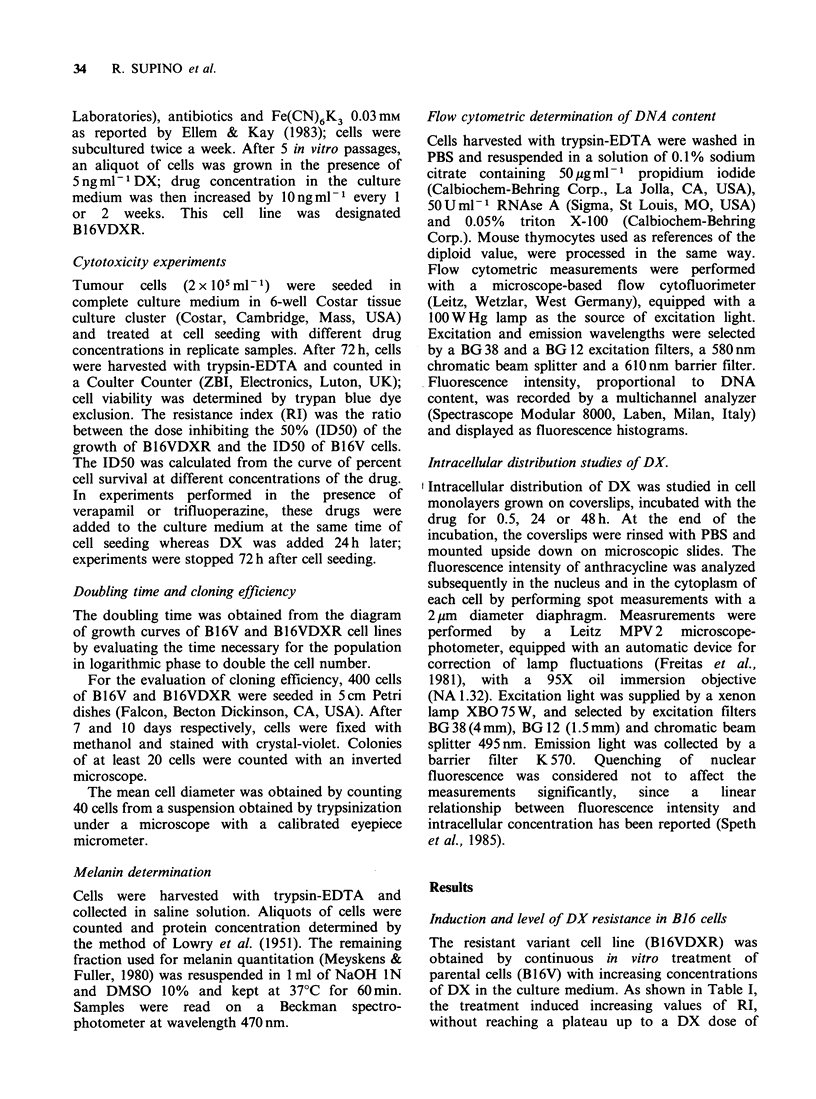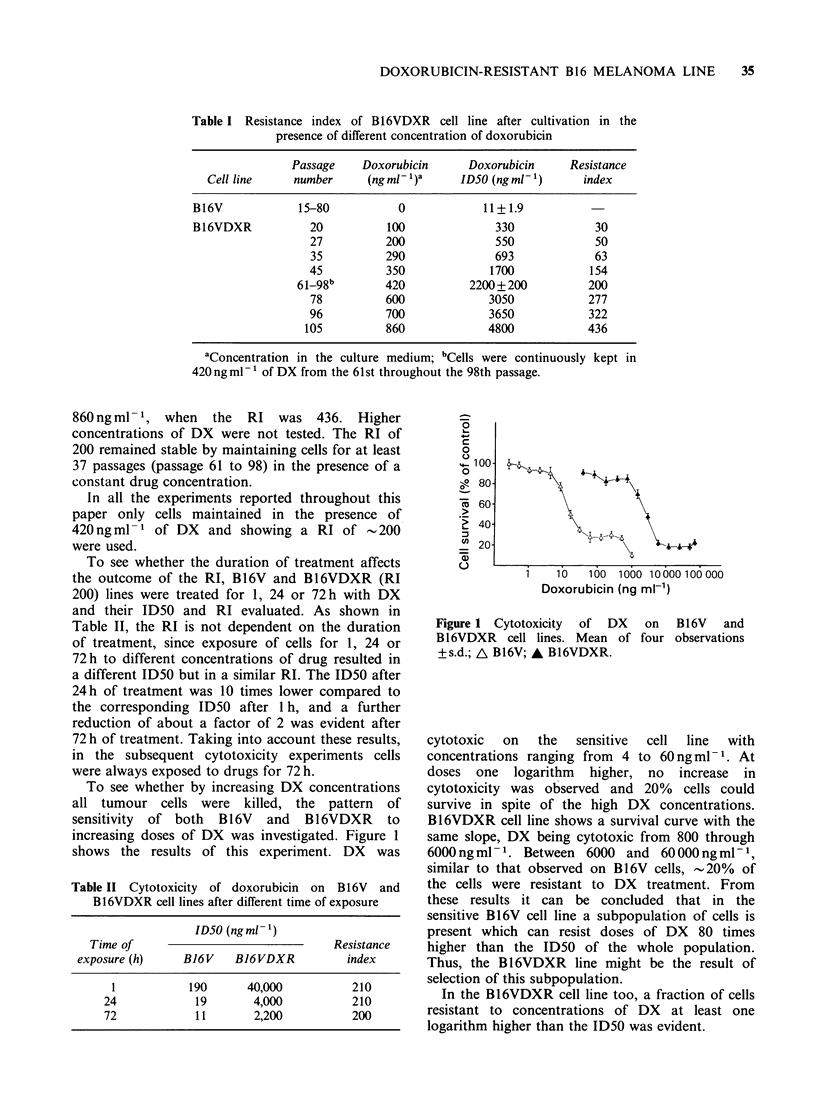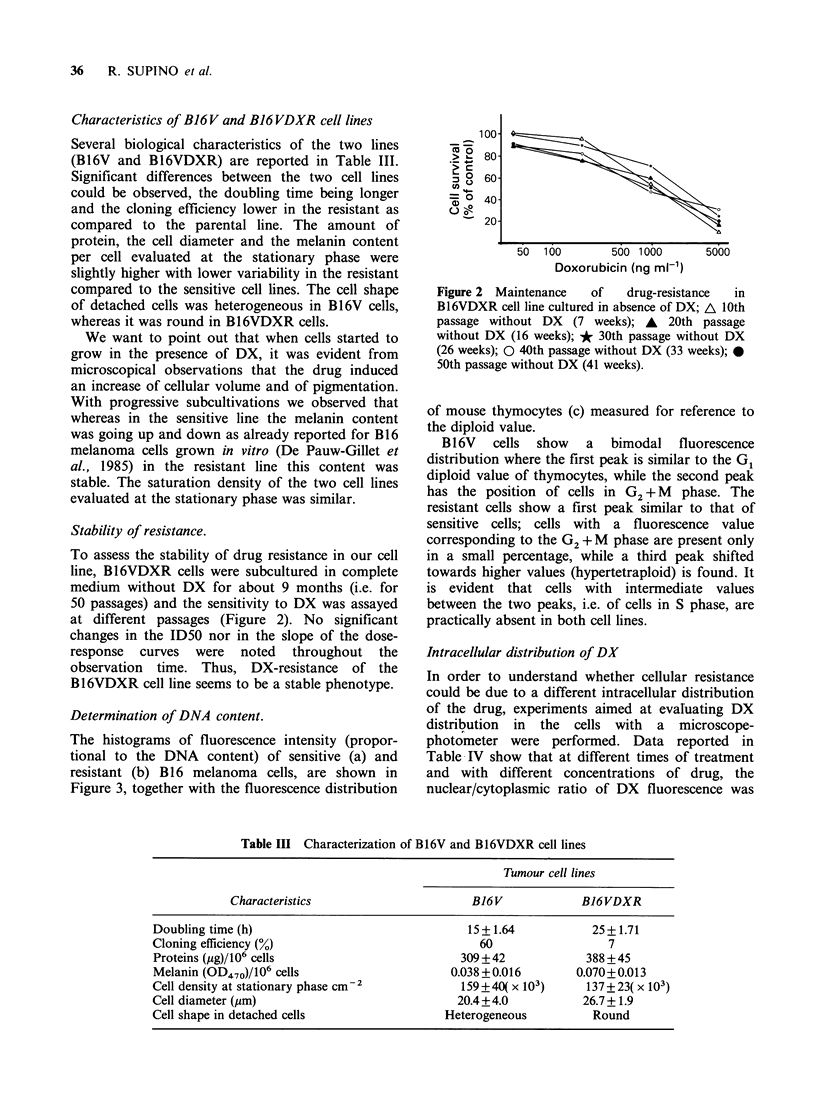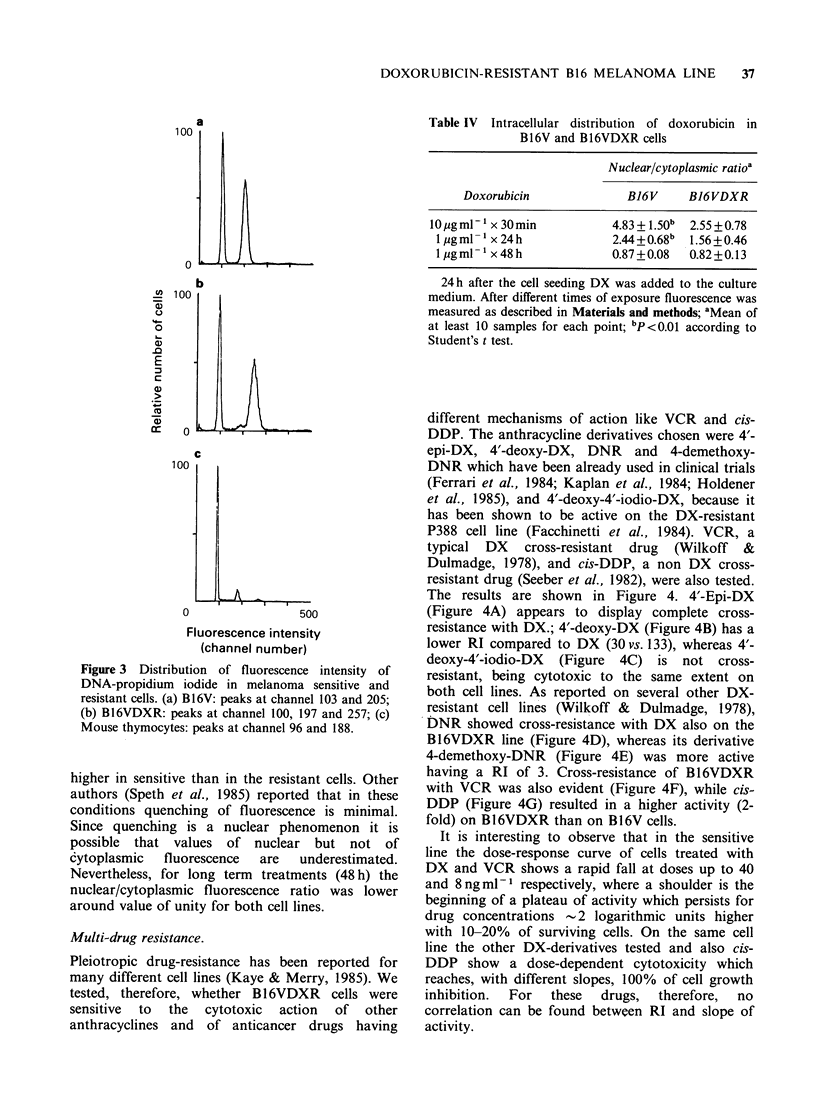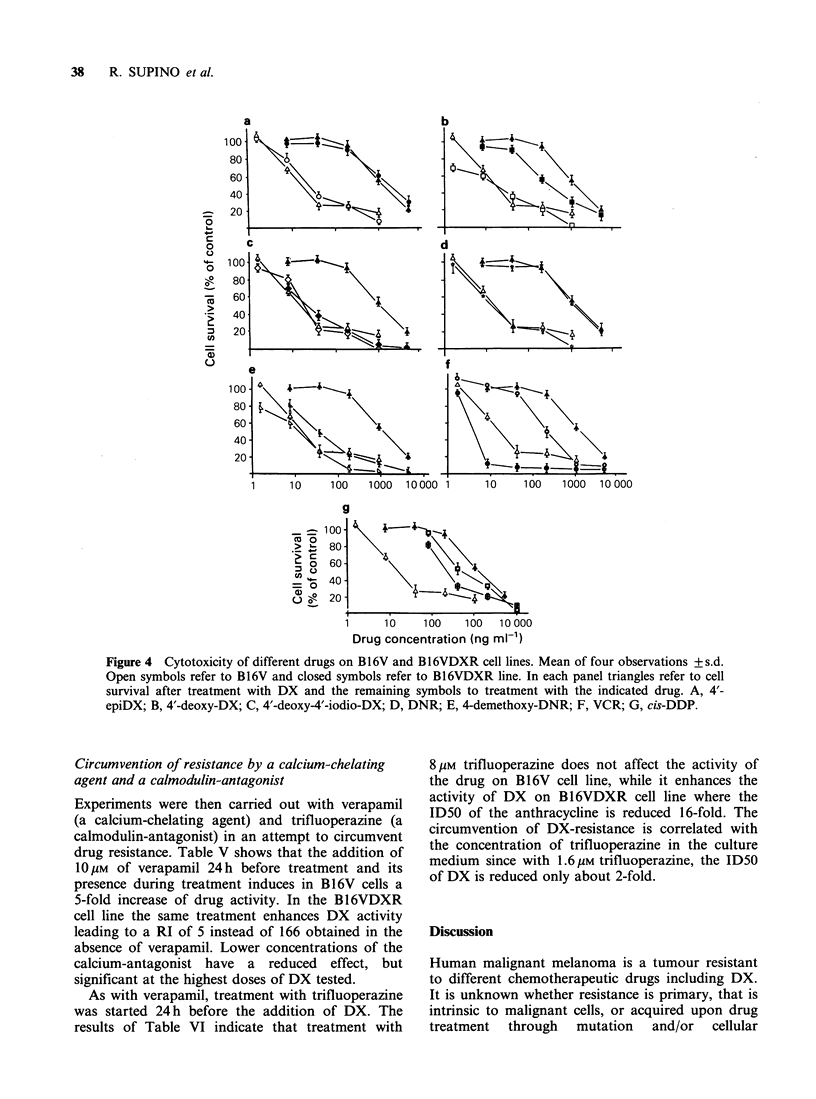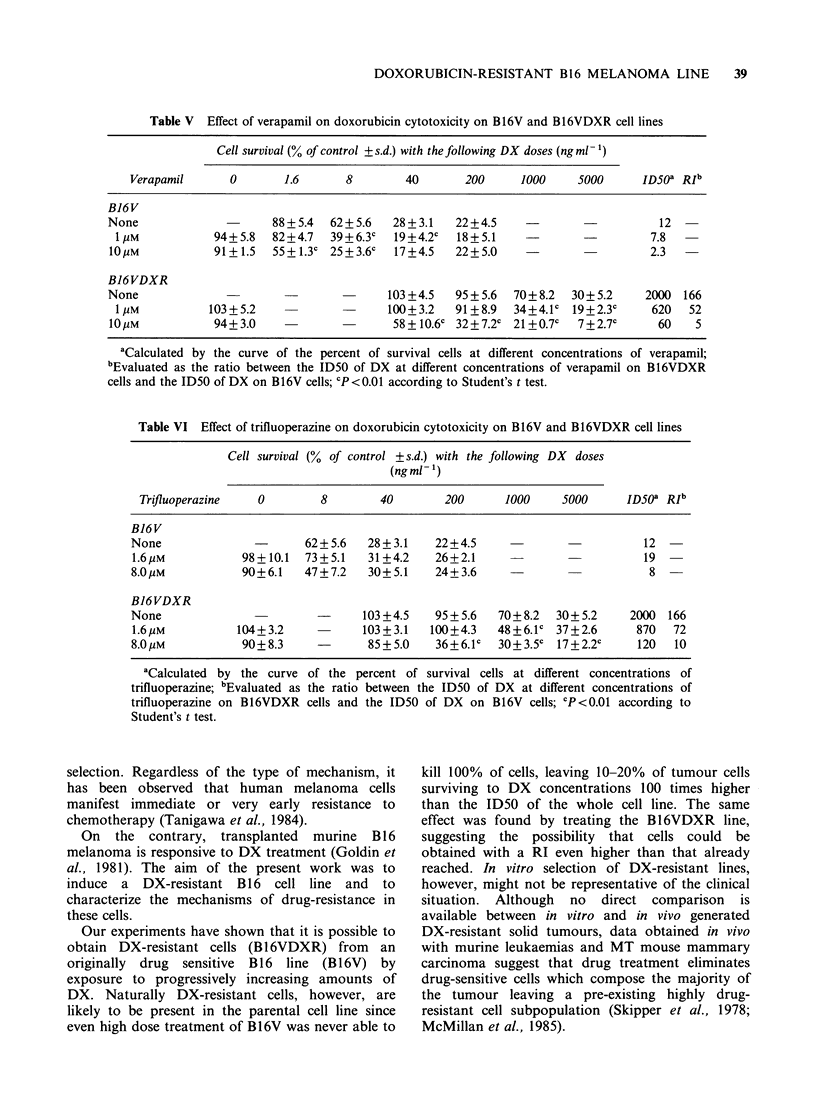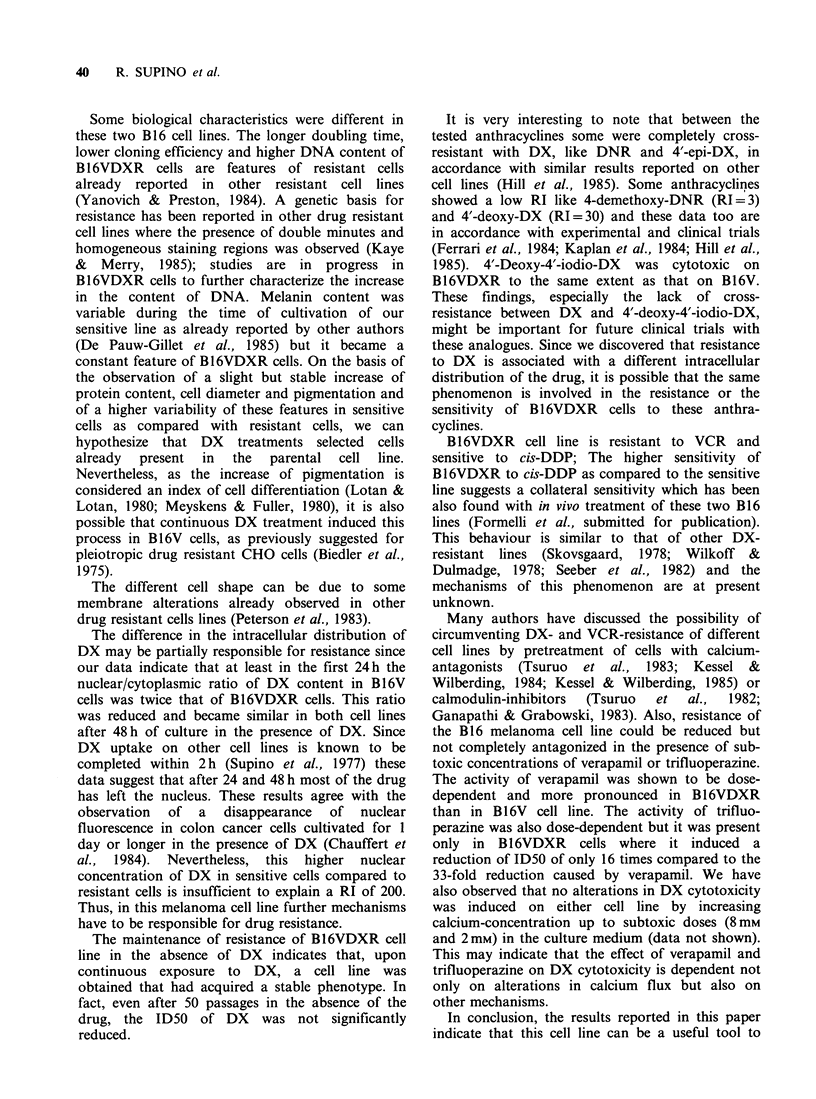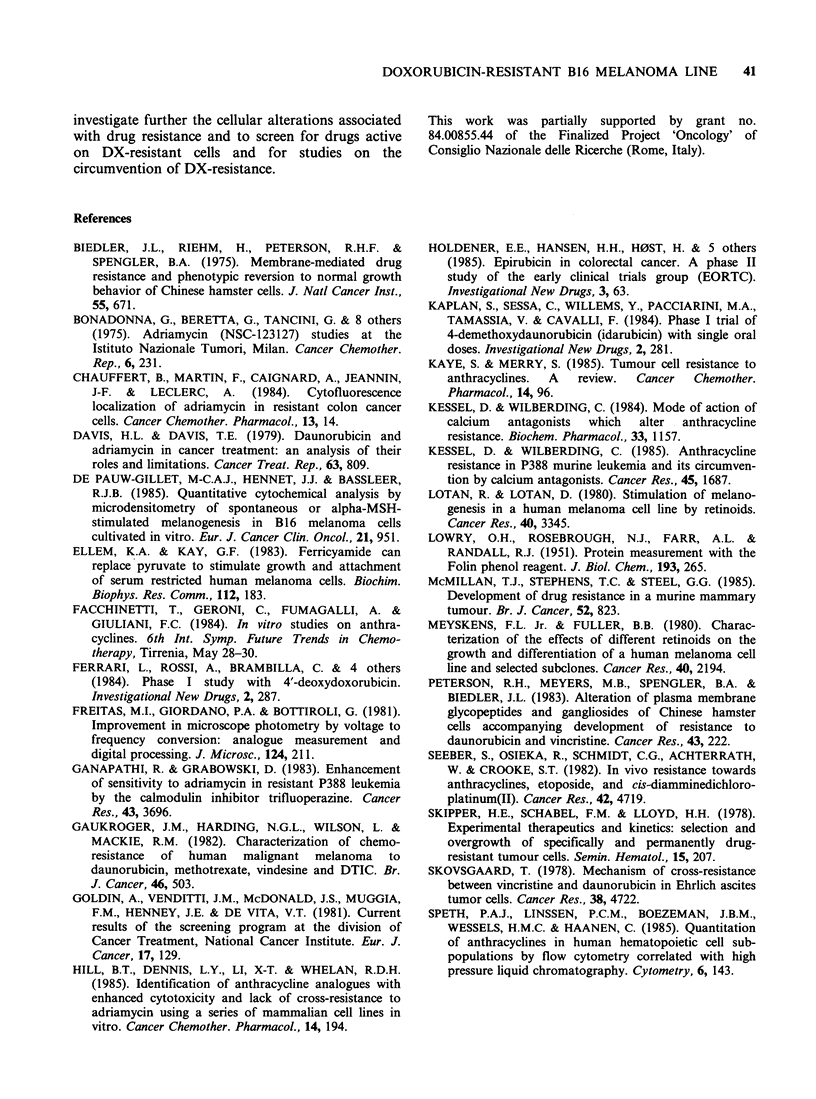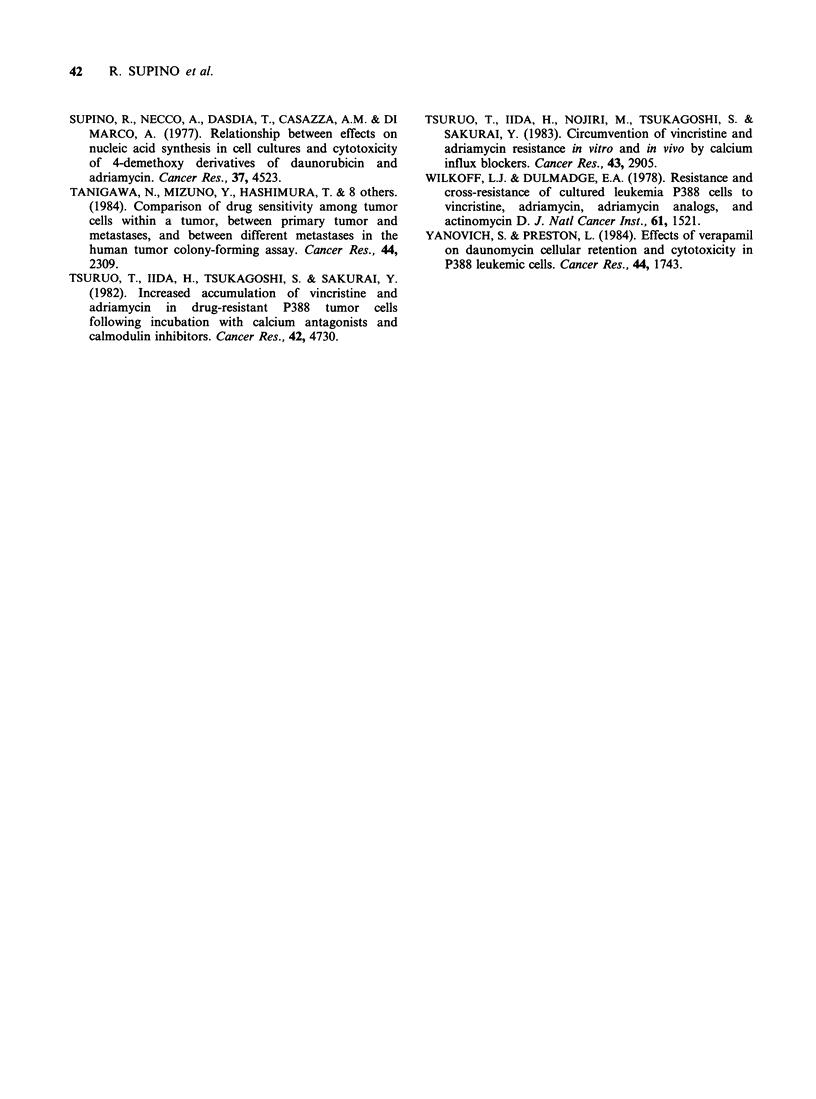# Characterization of a doxorubicin-resistant murine melanoma line: studies on cross-resistance and its circumvention.

**DOI:** 10.1038/bjc.1986.149

**Published:** 1986-07

**Authors:** R. Supino, E. Prosperi, F. Formelli, M. Mariani, G. Parmiani

## Abstract

A B16 mouse melanoma cell line resistant to doxorubicin was obtained by continuous in vitro exposure to the drug. The ID50 for this line was 200 times higher than that for the parental cell line. The resistant cell line had some biological characteristics similar to those of the sensitive parental cell line, like saturation density and protein content. Differences were found in doubling time which was longer, cloning efficiency which was lower and DNA content which was higher in the resistant as compared to the parental line. Intracellular distribution of doxorubicin was also different having a nuclear-cytoplasmic ratio higher in sensitive than in resistant cells. Melanin content was an unstable feature in the sensitive cell line, whereas melanin was always present in resistant cells. Resistance to doxorubicin was maintained during 50 in vitro passages in the absence of the drug. Cross-resistance was found with vincristine and other anthracyclines, like daunorubicin and 4'-epi-doxorubicin but not with cis-platinum, and a new doxorubicin derivative, 4'-deoxy-4'-iodio-doxorubicin. The B16 line showed a lower resistance index to 4'-deoxy-doxorubicin and 4-demethoxy-daunorubicin (30 and 3 respectively), as compared to doxorubicin. Doxorubicin-resistance was partially circumvented by pretreatment of resistant cells with verapamil, a calcium chelating agent, and by trifluoperazine, a calmodulin-antagonist.


Br. J. Cancer (1986), **54**, 33–42

### Characterization of a doxorubicin-resistant murine melanoma line: Studies on cross-resistance and its circumvention

R. Supino[1], E. Prosperi[2], F. Formelli[1], M. Mariani[1] & G. Parmiani[1]

[1]*Division of Experimental Oncology B, Istituto Nazionale per lo Studio e la Cura dei Tumori, Via Venezian 1, 20133 Milan and* [2]*Centro di Studio per l'Istochimica del CNR, Dipartimento di Biologia Animale, University of Pavia, Italy.*

**Summary** A B16 mouse melanoma cell line resistant to doxorubicin was obtained by continuous *in vitro* exposure to the drug. The ID50 for this line was 200 times higher than that for the parental cell line. The resistant cell line had some biological characteristics similar to those of the sensitive parental cell line, like saturation density and protein content. Differences were found in doubling time which was longer, cloning efficiency which was lower and DNA content which was higher in the resistant as compared to the parental line. Intracellular distribution of doxorubicin was also different having a nuclear-cytoplasmic ratio higher in sensitive than in resistant cells. Melanin content was an unstable feature in the sensitive cell line, whereas melanin was always present in resistant cells. Resistance to doxorubicin was maintained during 50 *in vitro* passages in the absence of the drug. Cross-resistance was found with vincristine and other anthracyclines, like daunorubicin and 4′-epi-doxorubicin but not with *cis*-platinum, and a new doxorubicin derivative, 4′-deoxy-4′-iodio-doxorubicin. The B16 line showed a lower resistance index to 4′-deoxy-doxorubicin and 4-demethoxy-daunorubicin (30 and 3 respectively), as compared to doxorubicin. Doxorubicin-resistance was partially circumvented by pretreatment of resistant cells with verapamil, a calcium chelating agent, and by trifluoperazine, a calmodulin-antagonist.

Doxorubicin (DX) is among the most widely used cytotoxic agents because it is active on different kinds of human tumours (Bonadonna *et al.*, 1975; Davis & Davis, 1979). One limitation in its use, however, is the emergence of drug-resistance in the tumours under treatment (Kaye & Merry, 1985). The development of resistance to drugs is a common clinical problem in the treatment of various cancers but the mechanisms responsible for its appearance are still not fully understood. It is also unknown whether the resistance is a characteristic of a cell subpopulation of the original tumour or it is induced by the treatment itself.

Human malignant melanoma is unresponsive to chemotherapy because of the emergence of sub-populations of resistant cells (Gaukroger *et al.*, 1982). On the contrary, the mouse melanoma B16 is sensitive to DX treatment (Goldin *et al.*, 1981). In order to study the mechanisms of drug resistance, we selected a cell line of B16 melanoma with elevated levels of resistance to DX by continuous *in vitro* exposure to increasing concentrations of the drug. The DX-resistant and DX-sensitive cell lines were then characterized for different biological parameters in order to understand the possible basis of their different chemosensitivity. A calcium chelating agent and a calmodulin antagonist were used to circumvent drug resistance which was also challenged with some DX analogs, vincristine (VCR) and *cis*-diamminedichloroplatinum II (*cis*-DDP).

### Materials and methods

### Drugs

DX, daunorubicin (DNR), 4′-epi-DX, 4′-deoxy-DX, 4′-deoxy-4′-iodio-DX, 4-demethoxy-DNR and *cis*-DDP were a gift from Farmitalia-Carlo Erba (Milan, Italy). VCR was purchased from Eli-Lilly (Indianapolis, IN, USA). Verapamil was purchased as Isoptin by Knoll AG Liestal (Switzerland); trifluoperazine was obtained by Maggioni (Milan, Italy). All drugs were dissolved in distilled water immediately before use.

### Cell lines

A B16 melanoma cell line (B16V) was obtained by mechanical disaggregation of a B16 melanoma tumour grown in syngeneic C57BL/6 mice. Cells were transferred in tissue culture in RPMI 1640 medium (Flow Laboratories, Irvine, Ayrshire, UK) containing 10% foetal calf serum (FCS) (Flow

Correspondence: R. Supino.
Received 2 January 1986; and in revised form, 28 February 1986.

© The Macmillan Press Ltd., 1986

Laboratories), antibiotics and $Fe(CN)_6K_3$ 0.03 mM as reported by Ellem & Kay (1983); cells were subcultured twice a week. After 5 *in vitro* passages, an aliquot of cells was grown in the presence of 5 ng ml$^{-1}$ DX; drug concentration in the culture medium was then increased by 10 ng ml$^{-1}$ every 1 or 2 weeks. This cell line was designated B16VDXR.

*Cytotoxicity experiments*

Tumour cells ($2 \times 10^5$ ml$^{-1}$) were seeded in complete culture medium in 6-well Costar tissue culture cluster (Costar, Cambridge, Mass, USA) and treated at cell seeding with different drug concentrations in replicate samples. After 72 h, cells were harvested with trypsin-EDTA and counted in a Coulter Counter (ZBI, Electronics, Luton, UK); cell viability was determined by trypan blue dye exclusion. The resistance index (RI) was the ratio between the dose inhibiting the 50% (ID50) of the growth of B16VDXR and the ID50 of B16V cells. The ID50 was calculated from the curve of percent cell survival at different concentrations of the drug. In experiments performed in the presence of verapamil or trifluoperazine, these drugs were added to the culture medium at the same time of cell seeding whereas DX was added 24 h later; experiments were stopped 72 h after cell seeding.

*Doubling time and cloning efficiency*

The doubling time was obtained from the diagram of growth curves of B16V and B16VDXR cell lines by evaluating the time necessary for the population in logarithmic phase to double the cell number.

For the evaluation of cloning efficiency, 400 cells of B16V and B16VDXR were seeded in 5 cm Petri dishes (Falcon, Becton Dickinson, CA, USA). After 7 and 10 days respectively, cells were fixed with methanol and stained with crystal-violet. Colonies of at least 20 cells were counted with an inverted microscope.

The mean cell diameter was obtained by counting 40 cells from a suspension obtained by trypsinization under a microscope with a calibrated eyepiece micrometer.

*Melanin determination*

Cells were harvested with trypsin-EDTA and collected in saline solution. Aliquots of cells were counted and protein concentration determined by the method of Lowry *et al.* (1951). The remaining fraction used for melanin quantitation (Meyskens & Fuller, 1980) was resuspended in 1 ml of NaOH 1N and DMSO 10% and kept at 37°C for 60 min. Samples were read on a Beckman spectrophotometer at wavelength 470 nm.

*Flow cytometric determination of DNA content*

Cells harvested with trypsin-EDTA were washed in PBS and resuspended in a solution of 0.1% sodium citrate containing 50 μg ml$^{-1}$ propidium iodide (Calbiochem-Behring Corp., La Jolla, CA, USA), 50 U ml$^{-1}$ RNAse A (Sigma, St Louis, MO, USA) and 0.05% triton X-100 (Calbiochem-Behring Corp.). Mouse thymocytes used as references of the diploid value, were processed in the same way. Flow cytometric measurements were performed with a microscope-based flow cytofluorimeter (Leitz, Wetzlar, West Germany), equipped with a 100 W Hg lamp as the source of excitation light. Excitation and emission wavelengths were selected by a BG 38 and a BG 12 excitation filters, a 580 nm chromatic beam splitter and a 610 nm barrier filter. Fluorescence intensity, proportional to DNA content, was recorded by a multichannel analyzer (Spectrascope Modular 8000, Laben, Milan, Italy) and displayed as fluorescence histograms.

*Intracellular distribution studies of DX.*

Intracellular distribution of DX was studied in cell monolayers grown on coverslips, incubated with the drug for 0.5, 24 or 48 h. At the end of the incubation, the coverslips were rinsed with PBS and mounted upside down on microscopic slides. The fluorescence intensity of anthracycline was analyzed subsequently in the nucleus and in the cytoplasm of each cell by performing spot measurements with a 2 μm diameter diaphragm. Measrurements were performed by a Leitz MPV 2 microscope-photometer, equipped with an automatic device for correction of lamp fluctuations (Freitas *et al.*, 1981), with a 95X oil immersion objective (NA 1.32). Excitation light was supplied by a xenon lamp XBO 75 W, and selected by excitation filters BG 38 (4 mm), BG 12 (1.5 mm) and chromatic beam splitter 495 nm. Emission light was collected by a barrier filter K 570. Quenching of nuclear fluorescence was considered not to affect the measurements significantly, since a linear relationship between fluorescence intensity and intracellular concentration has been reported (Speth *et al.*, 1985).

### Results

*Induction and level of DX resistance in B16 cells*

The resistant variant cell line (B16VDXR) was obtained by continuous *in vitro* treatment of parental cells (B16V) with increasing concentrations of DX in the culture medium. As shown in Table I, the treatment induced increasing values of RI, without reaching a plateau up to a DX dose of

**Table I** Resistance index of B16VDXR cell line after cultivation in the presence of different concentration of doxorubicin

| Cell line | *Passage number* | *Doxorubicin ($ng\,ml^{-1}$)*[a] | *Doxorubicin ID50 ($ng\,ml^{-1}$)* | *Resistance index* |
|---|---|---|---|---|
| B16V | 15–80 | 0 | $11 \pm 1.9$ | — |
| B16VDXR | 20 | 100 | 330 | 30 |
| | 27 | 200 | 550 | 50 |
| | 35 | 290 | 693 | 63 |
| | 45 | 350 | 1700 | 154 |
| | 61–98[b] | 420 | $2200 \pm 200$ | 200 |
| | 78 | 600 | 3050 | 277 |
| | 96 | 700 | 3650 | 322 |
| | 105 | 860 | 4800 | 436 |

[a]Concentration in the culture medium; [b]Cells were continuously kept in $420\,ng\,ml^{-1}$ of DX from the 61st throughout the 98th passage.

$860\,ng\,ml^{-1}$, when the RI was 436. Higher concentrations of DX were not tested. The RI of 200 remained stable by maintaining cells for at least 37 passages (passage 61 to 98) in the presence of a constant drug concentration.

In all the experiments reported throughout this paper only cells maintained in the presence of $420\,ng\,ml^{-1}$ of DX and showing a RI of $\sim 200$ were used.

To see whether the duration of treatment affects the outcome of the RI, B16V and B16VDXR (RI 200) lines were treated for 1, 24 or 72 h with DX and their ID50 and RI evaluated. As shown in Table II, the RI is not dependent on the duration of treatment, since exposure of cells for 1, 24 or 72 h to different concentrations of drug resulted in a different ID50 but in a similar RI. The ID50 after 24 h of treatment was 10 times lower compared to the corresponding ID50 after 1 h, and a further reduction of about a factor of 2 was evident after 72 h of treatment. Taking into account these results, in the subsequent cytotoxicity experiments cells were always exposed to drugs for 72 h.

To see whether by increasing DX concentrations all tumour cells were killed, the pattern of sensitivity of both B16V and B16VDXR to increasing doses of DX was investigated. Figure 1 shows the results of this experiment. DX was

**Table II** Cytotoxicity of doxorubicin on B16V and B16VDXR cell lines after different time of exposure

| *Time of exposure (h)* | *ID50 ($ng\,ml^{-1}$)* | | *Resistance index* |
|---|---|---|---|
| | *B16V* | *B16VDXR* | |
| 1 | 190 | 40,000 | 210 |
| 24 | 19 | 4,000 | 210 |
| 72 | 11 | 2,200 | 200 |

**Figure 1** Cytotoxicity of DX on B16V and B16VDXR cell lines. Mean of four observations ±s.d.; △ B16V; ▲ B16VDXR.

cytotoxic on the sensitive cell line with concentrations ranging from 4 to $60\,ng\,ml^{-1}$. At doses one logarithm higher, no increase in cytotoxicity was observed and 20% cells could survive in spite of the high DX concentrations. B16VDXR cell line shows a survival curve with the same slope, DX being cytotoxic from 800 through $6000\,ng\,ml^{-1}$. Between 6000 and $60\,000\,ng\,ml^{-1}$, similar to that observed on B16V cells, $\sim 20\%$ of the cells were resistant to DX treatment. From these results it can be concluded that in the sensitive B16V cell line a subpopulation of cells is present which can resist doses of DX 80 times higher than the ID50 of the whole population. Thus, the B16VDXR line might be the result of selection of this subpopulation.

In the B16VDXR cell line too, a fraction of cells resistant to concentrations of DX at least one logarithm higher than the ID50 was evident.

*Characteristics of B16V and B16VDXR cell lines*

Several biological characteristics of the two lines (B16V and B16VDXR) are reported in Table III. Significant differences between the two cell lines could be observed, the doubling time being longer and the cloning efficiency lower in the resistant as compared to the parental line. The amount of protein, the cell diameter and the melanin content per cell evaluated at the stationary phase were slightly higher with lower variability in the resistant compared to the sensitive cell lines. The cell shape of detached cells was heterogeneous in B16V cells, whereas it was round in B16VDXR cells.

We want to point out that when cells started to grow in the presence of DX, it was evident from microscopical observations that the drug induced an increase of cellular volume and of pigmentation. With progressive subcultivations we observed that whereas in the sensitive line the melanin content was going up and down as already reported for B16 melanoma cells grown *in vitro* (De Pauw-Gillet *et al.,* 1985) in the resistant line this content was stable. The saturation density of the two cell lines evaluated at the stationary phase was similar.

*Stability of resistance.*

To assess the stability of drug resistance in our cell line, B16VDXR cells were subcultured in complete medium without DX for about 9 months (i.e. for 50 passages) and the sensitivity to DX was assayed at different passages (Figure 2). No significant changes in the ID50 nor in the slope of the dose-response curves were noted throughout the observation time. Thus, DX-resistance of the B16VDXR cell line seems to be a stable phenotype.

*Determination of DNA content.*

The histograms of fluorescence intensity (proportional to the DNA content) of sensitive (a) and resistant (b) B16 melanoma cells, are shown in Figure 3, together with the fluorescence distribution of mouse thymocytes (c) measured for reference to the diploid value.

**Figure 2** Maintenance of drug-resistance in B16VDXR cell line cultured in absence of DX; △ 10th passage without DX (7 weeks); ▲ 20th passage without DX (16 weeks); ★ 30th passage without DX (26 weeks); ○ 40th passage without DX (33 weeks); ● 50th passage without DX (41 weeks).

B16V cells show a bimodal fluorescence distribution where the first peak is similar to the $G_1$ diploid value of thymocytes, while the second peak has the position of cells in $G_2+M$ phase. The resistant cells show a first peak similar to that of sensitive cells; cells with a fluorescence value corresponding to the $G_2+M$ phase are present only in a small percentage, while a third peak shifted towards higher values (hypertetraploid) is found. It is evident that cells with intermediate values between the two peaks, i.e. of cells in S phase, are practically absent in both cell lines.

*Intracellular distribution of DX*

In order to understand whether cellular resistance could be due to a different intracellular distribution of the drug, experiments aimed at evaluating DX distribution in the cells with a microscope-photometer were performed. Data reported in Table IV show that at different times of treatment and with different concentrations of drug, the nuclear/cytoplasmic ratio of DX fluorescence was

**Table III** Characterization of B16V and B16VDXR cell lines

| | *Tumour cell lines* | |
|---|---|---|
| *Characteristics* | *B16V* | *B16VDXR* |
| Doubling time (h) | 15 ± 1.64 | 25 ± 1.71 |
| Cloning efficiency (%) | 60 | 7 |
| Proteins (μg)/$10^6$ cells | 309 ± 42 | 388 ± 45 |
| Melanin ($OD_{470}$)/$10^6$ cells | 0.038 ± 0.016 | 0.070 ± 0.013 |
| Cell density at stationary phase cm$^{-2}$ | 159 ± 40( × $10^3$) | 137 ± 23( × $10^3$) |
| Cell diameter (μm) | 20.4 ± 4.0 | 26.7 ± 1.9 |
| Cell shape in detached cells | Heterogeneous | Round |

**Figure 3** Distribution of fluorescence intensity of DNA-propidium iodide in melanoma sensitive and resistant cells. (a) B16V: peaks at channel 103 and 205; (b) B16VDXR: peaks at channel 100, 197 and 257; (c) Mouse thymocytes: peaks at channel 96 and 188.

higher in sensitive than in the resistant cells. Other authors (Speth *et al.,* 1985) reported that in these conditions quenching of fluorescence is minimal. Since quenching is a nuclear phenomenon it is possible that values of nuclear but not of cytoplasmic fluorescence are underestimated. Nevertheless, for long term treatments (48 h) the nuclear/cytoplasmic fluorescence ratio was lower around value of unity for both cell lines.

*Multi-drug resistance.*

Pleiotropic drug-resistance has been reported for many different cell lines (Kaye & Merry, 1985). We tested, therefore, whether B16VDXR cells were sensitive to the cytotoxic action of other anthracyclines and of anticancer drugs having

**Table IV** Intracellular distribution of doxorubicin in B16V and B16VDXR cells

| | *Nuclear/cytoplasmic ratio*[a] | |
|---|---|---|
| *Doxorubicin* | *B16V* | *B16VDXR* |
| $10\,\mu g\,ml^{-1} \times 30\,min$ | $4.83 \pm 1.50$[b] | $2.55 \pm 0.78$ |
| $1\,\mu g\,ml^{-1} \times 24\,h$ | $2.44 \pm 0.68$[b] | $1.56 \pm 0.46$ |
| $1\,\mu g\,ml^{-1} \times 48\,h$ | $0.87 \pm 0.08$ | $0.82 \pm 0.13$ |

24 h after the cell seeding DX was added to the culture medium. After different times of exposure fluorescence was measured as described in **Materials and methods**; [a]Mean of at least 10 samples for each point; [b]$P < 0.01$ according to Student's *t* test.

different mechanisms of action like VCR and *cis*-DDP. The anthracycline derivatives chosen were 4′-epi-DX, 4′-deoxy-DX, DNR and 4-demethoxy-DNR which have been already used in clinical trials (Ferrari *et al.,* 1984; Kaplan *et al.,* 1984; Holdener *et al.,* 1985), and 4′-deoxy-4′-iodio-DX, because it has been shown to be active on the DX-resistant P388 cell line (Facchinetti *et al.,* 1984). VCR, a typical DX cross-resistant drug (Wilkoff & Dulmadge, 1978), and *cis*-DDP, a non DX cross-resistant drug (Seeber *et al.,* 1982), were also tested. The results are shown in Figure 4. 4′-Epi-DX (Figure 4A) appears to display complete cross-resistance with DX.; 4′-deoxy-DX (Figure 4B) has a lower RI compared to DX (30 *vs.* 133), whereas 4′-deoxy-4′-iodio-DX (Figure 4C) is not cross-resistant, being cytotoxic to the same extent on both cell lines. As reported on several other DX-resistant cell lines (Wilkoff & Dulmadge, 1978), DNR showed cross-resistance with DX also on the B16VDXR line (Figure 4D), whereas its derivative 4-demethoxy-DNR (Figure 4E) was more active having a RI of 3. Cross-resistance of B16VDXR with VCR was also evident (Figure 4F), while *cis*-DDP (Figure 4G) resulted in a higher activity (2-fold) on B16VDXR than on B16V cells.

It is interesting to observe that in the sensitive line the dose-response curve of cells treated with DX and VCR shows a rapid fall at doses up to 40 and $8\,ng\,ml^{-1}$ respectively, where a shoulder is the beginning of a plateau of activity which persists for drug concentrations ~2 logarithmic units higher with 10–20% of surviving cells. On the same cell line the other DX-derivatives tested and also *cis*-DDP show a dose-dependent cytotoxicity which reaches, with different slopes, 100% of cell growth inhibition. For these drugs, therefore, no correlation can be found between RI and slope of activity.

**Figure 4** Cytotoxicity of different drugs on B16V and B16VDXR cell lines. Mean of four observations ±s.d. Open symbols refer to B16V and closed symbols refer to B16VDXR line. In each panel triangles refer to cell survival after treatment with DX and the remaining symbols to treatment with the indicated drug. A, 4′-epiDX; B, 4′-deoxy-DX; C, 4′-deoxy-4′-iodio-DX; D, DNR; E, 4-demethoxy-DNR; F, VCR; G, *cis*-DDP.

*Circumvention of resistance by a calcium-chelating agent and a calmodulin-antagonist*

Experiments were then carried out with verapamil (a calcium-chelating agent) and trifluoperazine (a calmodulin-antagonist) in an attempt to circumvent drug resistance. Table V shows that the addition of 10 μM of verapamil 24 h before treatment and its presence during treatment induces in B16V cells a 5-fold increase of drug activity. In the B16VDXR cell line the same treatment enhances DX activity leading to a RI of 5 instead of 166 obtained in the absence of verapamil. Lower concentrations of the calcium-antagonist have a reduced effect, but significant at the highest doses of DX tested.

As with verapamil, treatment with trifluoperazine was started 24 h before the addition of DX. The results of Table VI indicate that treatment with 8 μM trifluoperazine does not affect the activity of the drug on B16V cell line, while it enhances the activity of DX on B16VDXR cell line where the ID50 of the anthracycline is reduced 16-fold. The circumvention of DX-resistance is correlated with the concentration of trifluoperazine in the culture medium since with 1.6 μM trifluoperazine, the ID50 of DX is reduced only about 2-fold.

### Discussion

Human malignant melanoma is a tumour resistant to different chemotherapeutic drugs including DX. It is unknown whether resistance is primary, that is intrinsic to malignant cells, or acquired upon drug treatment through mutation and/or cellular

**Table V** Effect of verapamil on doxorubicin cytotoxicity on B16V and B16VDXR cell lines

| | *Cell survival (% of control ±s.d.) with the following DX doses (ng ml⁻¹)* | | | | | | | | |
|---|---|---|---|---|---|---|---|---|---|
| *Verapamil* | *0* | *1.6* | *8* | *40* | *200* | *1000* | *5000* | *ID50*[a] | *RI*[b] |
| *B16V* | | | | | | | | | |
| None | — | 88±5.4 | 62±5.6 | 28±3.1 | 22±4.5 | — | — | 12 | — |
| 1 μM | 94±5.8 | 82±4.7 | 39±6.3[c] | 19±4.2[c] | 18±5.1 | — | — | 7.8 | — |
| 10 μM | 91±1.5 | 55±1.3[c] | 25±3.6[c] | 17±4.5 | 22±5.0 | — | — | 2.3 | — |
| *B16VDXR* | | | | | | | | | |
| None | — | — | — | 103±4.5 | 95±5.6 | 70±8.2 | 30±5.2 | 2000 | 166 |
| 1 μM | 103±5.2 | — | — | 100±3.2 | 91±8.9 | 34±4.1[c] | 19±2.3[c] | 620 | 52 |
| 10 μM | 94±3.0 | — | — | 58±10.6[c] | 32±7.2[c] | 21±0.7[c] | 7±2.7[c] | 60 | 5 |

[a]Calculated by the curve of the percent of survival cells at different concentrations of verapamil; [b]Evaluated as the ratio between the ID50 of DX at different concentrations of verapamil on B16VDXR cells and the ID50 of DX on B16V cells; [c]$P<0.01$ according to Student's *t* test.

**Table VI** Effect of trifluoperazine on doxorubicin cytotoxicity on B16V and B16VDXR cell lines

| | *Cell survival (% of control ±s.d.) with the following DX doses (ng ml⁻¹)* | | | | | | | |
|---|---|---|---|---|---|---|---|---|
| *Trifluoperazine* | *0* | *8* | *40* | *200* | *1000* | *5000* | *ID50*[a] | *RI*[b] |
| *B16V* | | | | | | | | |
| None | — | 62±5.6 | 28±3.1 | 22±4.5 | — | — | 12 | — |
| 1.6 μM | 98±10.1 | 73±5.1 | 31±4.2 | 26±2.1 | — | — | 19 | — |
| 8.0 μM | 90±6.1 | 47±7.2 | 30±5.1 | 24±3.6 | — | — | 8 | — |
| *B16VDXR* | | | | | | | | |
| None | — | — | 103±4.5 | 95±5.6 | 70±8.2 | 30±5.2 | 2000 | 166 |
| 1.6 μM | 104±3.2 | — | 103±3.1 | 100±4.3 | 48±6.1[c] | 37±2.6 | 870 | 72 |
| 8.0 μM | 90±8.3 | — | 85±5.0 | 36±6.1[c] | 30±3.5[c] | 17±2.2[c] | 120 | 10 |

[a]Calculated by the curve of the percent of survival cells at different concentrations of trifluoperazine; [b]Evaluated as the ratio between the ID50 of DX at different concentrations of trifluoperazine on B16VDXR cells and the ID50 of DX on B16V cells; [c]$P<0.01$ according to Student's *t* test.

selection. Regardless of the type of mechanism, it has been observed that human melanoma cells manifest immediate or very early resistance to chemotherapy (Tanigawa *et al.*, 1984).

On the contrary, transplanted murine B16 melanoma is responsive to DX treatment (Goldin *et al.*, 1981). The aim of the present work was to induce a DX-resistant B16 cell line and to characterize the mechanisms of drug-resistance in these cells.

Our experiments have shown that it is possible to obtain DX-resistant cells (B16VDXR) from an originally drug sensitive B16 line (B16V) by exposure to progressively increasing amounts of DX. Naturally DX-resistant cells, however, are likely to be present in the parental cell line since even high dose treatment of B16V was never able to kill 100% of cells, leaving 10–20% of tumour cells surviving to DX concentrations 100 times higher than the ID50 of the whole cell line. The same effect was found by treating the B16VDXR line, suggesting the possibility that cells could be obtained with a RI even higher than that already reached. *In vitro* selection of DX-resistant lines, however, might not be representative of the clinical situation. Although no direct comparison is available between *in vitro* and *in vivo* generated DX-resistant solid tumours, data obtained *in vivo* with murine leukaemias and MT mouse mammary carcinoma suggest that drug treatment eliminates drug-sensitive cells which compose the majority of the tumour leaving a pre-existing highly drug-resistant cell subpopulation (Skipper *et al.*, 1978; McMillan *et al.*, 1985).

Some biological characteristics were different in these two B16 cell lines. The longer doubling time, lower cloning efficiency and higher DNA content of B16VDXR cells are features of resistant cells already reported in other resistant cell lines (Yanovich & Preston, 1984). A genetic basis for resistance has been reported in other drug resistant cell lines where the presence of double minutes and homogeneous staining regions was observed (Kaye & Merry, 1985); studies are in progress in B16VDXR cells to further characterize the increase in the content of DNA. Melanin content was variable during the time of cultivation of our sensitive line as already reported by other authors (De Pauw-Gillet *et al.*, 1985) but it became a constant feature of B16VDXR cells. On the basis of the observation of a slight but stable increase of protein content, cell diameter and pigmentation and of a higher variability of these features in sensitive cells as compared with resistant cells, we can hypothesize that DX treatments selected cells already present in the parental cell line. Nevertheless, as the increase of pigmentation is considered an index of cell differentiation (Lotan & Lotan, 1980; Meyskens & Fuller, 1980), it is also possible that continuous DX treatment induced this process in B16V cells, as previously suggested for pleiotropic drug resistant CHO cells (Biedler *et al.*, 1975).

The different cell shape can be due to some membrane alterations already observed in other drug resistant cells lines (Peterson *et al.*, 1983).

The difference in the intracellular distribution of DX may be partially responsible for resistance since our data indicate that at least in the first 24 h the nuclear/cytoplasmic ratio of DX content in B16V cells was twice that of B16VDXR cells. This ratio was reduced and became similar in both cell lines after 48 h of culture in the presence of DX. Since DX uptake on other cell lines is known to be completed within 2 h (Supino *et al.*, 1977) these data suggest that after 24 and 48 h most of the drug has left the nucleus. These results agree with the observation of a disappearance of nuclear fluorescence in colon cancer cells cultivated for 1 day or longer in the presence of DX (Chauffert *et al.*, 1984). Nevertheless, this higher nuclear concentration of DX in sensitive cells compared to resistant cells is insufficient to explain a RI of 200. Thus, in this melanoma cell line further mechanisms have to be responsible for drug resistance.

The maintenance of resistance of B16VDXR cell line in the absence of DX indicates that, upon continuous exposure to DX, a cell line was obtained that had acquired a stable phenotype. In fact, even after 50 passages in the absence of the drug, the ID50 of DX was not significantly reduced.

It is very interesting to note that between the tested anthracyclines some were completely cross-resistant with DX, like DNR and 4′-epi-DX, in accordance with similar results reported on other cell lines (Hill *et al.*, 1985). Some anthracyclines showed a low RI like 4-demethoxy-DNR (RI = 3) and 4′-deoxy-DX (RI = 30) and these data too are in accordance with experimental and clinical trials (Ferrari *et al.*, 1984; Kaplan *et al.*, 1984; Hill *et al.*, 1985). 4′-Deoxy-4′-iodio-DX was cytotoxic on B16VDXR to the same extent as that on B16V. These findings, especially the lack of cross-resistance between DX and 4′-deoxy-4′-iodio-DX, might be important for future clinical trials with these analogues. Since we discovered that resistance to DX is associated with a different intracellular distribution of the drug, it is possible that the same phenomenon is involved in the resistance or the sensitivity of B16VDXR cells to these anthracyclines.

B16VDXR cell line is resistant to VCR and sensitive to *cis*-DDP; The higher sensitivity of B16VDXR to *cis*-DDP as compared to the sensitive line suggests a collateral sensitivity which has been also found with *in vivo* treatment of these two B16 lines (Formelli *et al.*, submitted for publication). This behaviour is similar to that of other DX-resistant lines (Skovsgaard, 1978; Wilkoff & Dulmadge, 1978; Seeber *et al.*, 1982) and the mechanisms of this phenomenon are at present unknown.

Many authors have discussed the possibility of circumventing DX- and VCR-resistance of different cell lines by pretreatment of cells with calcium-antagonists (Tsuruo *et al.*, 1983; Kessel & Wilberding, 1984; Kessel & Wilberding, 1985) or calmodulin-inhibitors (Tsuruo *et al.*, 1982; Ganapathi & Grabowski, 1983). Also, resistance of the B16 melanoma cell line could be reduced but not completely antagonized in the presence of subtoxic concentrations of verapamil or trifluoperazine. The activity of verapamil was shown to be dose-dependent and more pronounced in B16VDXR than in B16V cell line. The activity of trifluoperazine was also dose-dependent but it was present only in B16VDXR cells where it induced a reduction of ID50 of only 16 times compared to the 33-fold reduction caused by verapamil. We have also observed that no alterations in DX cytotoxicity was induced on either cell line by increasing calcium-concentration up to subtoxic doses (8 mM and 2 mM) in the culture medium (data not shown). This may indicate that the effect of verapamil and trifluoperazine on DX cytotoxicity is dependent not only on alterations in calcium flux but also on other mechanisms.

In conclusion, the results reported in this paper indicate that this cell line can be a useful tool to investigate further the cellular alterations associated with drug resistance and to screen for drugs active on DX-resistant cells and for studies on the circumvention of DX-resistance.

This work was partially supported by grant no. 84.00855.44 of the Finalized Project 'Oncology' of Consiglio Nazionale delle Ricerche (Rome, Italy).

### References

BIEDLER, J.L., RIEHM, H., PETERSON, R.H.F. & SPENGLER, B.A. (1975). Membrane-mediated drug resistance and phenotypic reversion to normal growth behavior of Chinese hamster cells. *J. Natl Cancer Inst.*, **55**, 671.

BONADONNA, G., BERETTA, G., TANCINI, G. & 8 others (1975). Adriamycin (NSC-123127) studies at the Istituto Nazionale Tumori, Milan. *Cancer Chemother. Rep.*, **6**, 231.

CHAUFFERT, B., MARTIN, F., CAIGNARD, A., JEANNIN, J-F. & LECLERC, A. (1984). Cytofluorescence localization of adriamycin in resistant colon cancer cells. *Cancer Chemother. Pharmacol.*, **13**, 14.

DAVIS, H.L. & DAVIS, T.E. (1979). Daunorubicin and adriamycin in cancer treatment: an analysis of their roles and limitations. *Cancer Treat. Rep.*, **63**, 809.

DE PAUW-GILLET, M-C.A.J., HENNET, J.J. & BASSLEER, R.J.B. (1985). Quantitative cytochemical analysis by microdensitometry of spontaneous or alpha-MSH-stimulated melanogenesis in B16 melanoma cells cultivated in vitro. *Eur. J. Cancer Clin. Oncol.*, **21**, 951.

ELLEM, K.A. & KAY, G.F. (1983). Ferricyamide can replace pyruvate to stimulate growth and attachment of serum restricted human melanoma cells. *Biochim. Biophys. Res. Comm.*, **112**, 183.

FACCHINETTI, T., GERONI, C., FUMAGALLI, A. & GIULIANI, F.C. (1984). *In vitro* studies on anthracyclines. *6th Int. Symp. Future Trends in Chemotherapy*, Tirrenia, May 28–30.

FERRARI, L., ROSSI, A., BRAMBILLA, C. & 4 others (1984). Phase I study with 4′-deoxydoxorubicin. *Investigational New Drugs*, **2**, 287.

FREITAS, M.I., GIORDANO, P.A. & BOTTIROLI, G. (1981). Improvement in microscope photometry by voltage to frequency conversion: analogue measurement and digital processing. *J. Microsc.*, **124**, 211.

GANAPATHI, R. & GRABOWSKI, D. (1983). Enhancement of sensitivity to adriamycin in resistant P388 leukemia by the calmodulin inhibitor trifluoperazine. *Cancer Res.*, **43**, 3696.

GAUKROGER, J.M., HARDING, N.G.L., WILSON, L. & MACKIE, R.M. (1982). Characterization of chemoresistance of human malignant melanoma to daunorubicin, methotrexate, vindesine and DTIC. *Br. J. Cancer*, **46**, 503.

GOLDIN, A., VENDITTI, J.M., McDONALD, J.S., MUGGIA, F.M., HENNEY, J.E. & DE VITA, V.T. (1981). Current results of the screening program at the division of Cancer Treatment, National Cancer Institute. *Eur. J. Cancer*, **17**, 129.

HILL, B.T., DENNIS, L.Y., LI, X-T. & WHELAN, R.D.H. (1985). Identification of anthracycline analogues with enhanced cytotoxicity and lack of cross-resistance to adriamycin using a series of mammalian cell lines in vitro. *Cancer Chemother. Pharmacol.*, **14**, 194.

HOLDENER, E.E., HANSEN, H.H., HØST, H. & 5 others (1985). Epirubicin in colorectal cancer. A phase II study of the early clinical trials group (EORTC). *Investigational New Drugs*, **3**, 63.

KAPLAN, S., SESSA, C., WILLEMS, Y., PACCIARINI, M.A., TAMASSIA, V. & CAVALLI, F. (1984). Phase I trial of 4-demethoxydaunorubicin (idarubicin) with single oral doses. *Investigational New Drugs*, **2**, 281.

KAYE, S. & MERRY, S. (1985). Tumour cell resistance to anthracyclines. A review. *Cancer Chemother. Pharmacol.*, **14**, 96.

KESSEL, D. & WILBERDING, C. (1984). Mode of action of calcium antagonists which alter anthracycline resistance. *Biochem. Pharmacol.*, **33**, 1157.

KESSEL, D. & WILBERDING, C. (1985). Anthracycline resistance in P388 murine leukemia and its circumvention by calcium antagonists. *Cancer Res.*, **45**, 1687.

LOTAN, R. & LOTAN, D. (1980). Stimulation of melanogenesis in a human melanoma cell line by retinoids. *Cancer Res.*, **40**, 3345.

LOWRY, O.H., ROSEBROUGH, N.J., FARR, A.L. & RANDALL, R.J. (1951). Protein measurement with the Folin phenol reagent. *J. Biol. Chem.*, **193**, 265.

McMILLAN, T.J., STEPHENS, T.C. & STEEL, G.G. (1985). Development of drug resistance in a murine mammary tumour. *Br. J. Cancer*, **52**, 823.

MEYSKENS, F.L. Jr. & FULLER, B.B. (1980). Characterization of the effects of different retinoids on the growth and differentiation of a human melanoma cell line and selected subclones. *Cancer Res.*, **40**, 2194.

PETERSON, R.H., MEYERS, M.B., SPENGLER, B.A. & BIEDLER, J.L. (1983). Alteration of plasma membrane glycopeptides and gangliosides of Chinese hamster cells accompanying development of resistance to daunorubicin and vincristine. *Cancer Res.*, **43**, 222.

SEEBER, S., OSIEKA, R., SCHMIDT, C.G., ACHTERRATH, W. & CROOKE, S.T. (1982). In vivo resistance towards anthracyclines, etoposide, and *cis*-diamminedichloroplatinum(II). *Cancer Res.*, **42**, 4719.

SKIPPER, H.E., SCHABEL, F.M. & LLOYD, H.H. (1978). Experimental therapeutics and kinetics: selection and overgrowth of specifically and permanently drug-resistant tumour cells. *Semin. Hematol.*, **15**, 207.

SKOVSGAARD, T. (1978). Mechanism of cross-resistance between vincristine and daunorubicin in Ehrlich ascites tumor cells. *Cancer Res.*, **38**, 4722.

SPETH, P.A.J., LINSSEN, P.C.M., BOEZEMAN, J.B.M., WESSELS, H.M.C. & HAANEN, C. (1985). Quantitation of anthracyclines in human hematopoietic cell subpopulations by flow cytometry correlated with high pressure liquid chromatography. *Cytometry*, **6**, 143.

SUPINO, R., NECCO, A., DASDIA, T., CASAZZA, A.M. & DI MARCO, A. (1977). Relationship between effects on nucleic acid synthesis in cell cultures and cytotoxicity of 4-demethoxy derivatives of daunorubicin and adriamycin. *Cancer Res.*, **37**, 4523.

TANIGAWA, N., MIZUNO, Y., HASHIMURA, T. & 8 others. (1984). Comparison of drug sensitivity among tumor cells within a tumor, between primary tumor and metastases, and between different metastases in the human tumor colony-forming assay. *Cancer Res.*, **44**, 2309.

TSURUO, T., IIDA, H., TSUKAGOSHI, S. & SAKURAI, Y. (1982). Increased accumulation of vincristine and adriamycin in drug-resistant P388 tumor cells following incubation with calcium antagonists and calmodulin inhibitors. *Cancer Res.*, **42**, 4730.

TSURUO, T., IIDA, H., NOJIRI, M., TSUKAGOSHI, S. & SAKURAI, Y. (1983). Circumvention of vincristine and adriamycin resistance *in vitro* and *in vivo* by calcium influx blockers. *Cancer Res.*, **43**, 2905.

WILKOFF, L.J. & DULMADGE, E.A. (1978). Resistance and cross-resistance of cultured leukemia P388 cells to vincristine, adriamycin, adriamycin analogs, and actinomycin D. *J. Natl Cancer Inst.*, **61**, 1521.

YANOVICH, S. & PRESTON, L. (1984). Effects of verapamil on daunomycin cellular retention and cytotoxicity in P388 leukemic cells. *Cancer Res.*, **44**, 1743.